# Roles of leptin in the recovery of muscle and bone by reloading after mechanical unloading in high fat diet-fed obese mice

**DOI:** 10.1371/journal.pone.0224403

**Published:** 2019-10-24

**Authors:** Naoyuki Kawao, Masayoshi Ishida, Hiroshi Kaji

**Affiliations:** Department of Physiology and Regenerative Medicine, Kindai University Faculty of Medicine, Osakasayama, Japan; East Tennessee State University, UNITED STATES

## Abstract

Muscle and bone masses are elevated by the increased mechanical stress associated with body weight gain in obesity. However, the mechanisms by which obesity affects muscle and bone remain unclear. We herein investigated the roles of obesity and humoral factors from adipose tissue in the recovery phase after reloading from disuse-induced muscle wasting and bone loss using normal diet (ND)- or high fat diet (HFD)-fed mice with hindlimb unloading (HU) and subsequent reloading. Obesity did not affect decreases in trabecular bone mineral density (BMD), muscle mass in the lower leg, or grip strength in HU mice. Obesity significantly increased trabecular BMD, muscle mass in the lower leg, and grip strength in reloading mice over those in reloading mice fed ND. Among the humoral factors in epididymal and subcutaneous adipose tissue, leptin mRNA levels were significantly higher in reloading mice fed HFD than in mice fed ND. Moreover, circulating leptin levels were significantly higher in reloading mice fed HFD than in mice fed ND. Leptin mRNA levels in epididymal adipose tissue or serum leptin levels positively correlated with the increases in trabecular BMD, total muscle mass, and grip strength in reloading mice fed ND and HFD. The present study is the first to demonstrate that obesity enhances the recovery of bone and muscle masses as well as strength decreased by disuse after reloading in mice. Leptin may contribute to the recovery of muscle and bone enhanced by obesity in mice.

## Introduction

Increasing evidence suggests that obesity affects bone metabolism and muscle functions [1–3]. Obese individuals have a higher bone mineral density (BMD) than non-obese individuals [4]. De Laet et al. revealed that obesity reduced self-reported overall and hip fracture risks in a meta-analysis [5]. On the other hand, Compston et al. reported that obesity is a risk factor for ankle and upper leg fractures in postmenopausal women, suggesting that obesity differently affects bone metabolism by the sites [6]. Moreover, in obese mice, tibial bone mass is increased by enhancing mechanical stress associated with body weight gain, but subsequently reduced by impairing bone metabolism [7]. Viljakainen et al. revealed that indices of bone metabolism are lower in obese individuals than in non-obese individuals, suggesting that obesity reduces bone turnover [8]. These findings indicate that the effects of obesity on bone metabolism are influenced by bone formation enhanced by mechanical stress and adipose tissue-derived abnormalities in bone metabolism.

Regarding skeletal muscle, obesity increases muscle mass and function in adolescent girls because increased weight-bearing played as a chronic mechanical loading on skeletal muscle [9]. In contrast, obesity reduces muscle mass and function in the elderly with sarcopenia [2]. Moreover, previous studies showed that obesity impairs myogenic differentiation in mice and reduces contractile function in skeletal muscle collected from mice [3,10]. Overall, these findings suggest that muscle mass is regulated by the balance of a training effect associated with body weight gain and a negative effect associated with metabolic abnormalities in obesity. However, the mechanisms by which obesity influences muscle and bone remain unclear.

White adipose tissue (WAT) affects other tissues through the release of humoral factors [11]. Adipocytokines, such as tumor necrosis factor (TNF)-α, plasminogen activator inhibitor (PAI)-1, monocyte chemoattractant protein (MCP)-1, leptin, and adiponectin, play critical roles in obesity-related diseases [11]. Among them, TNF-α, PAI-1, and MCP-1 are primarily recognized as negative regulators of muscle and bone [12–14]. Circulating adiponectin levels were found to be lower in obese individuals than in non-obese individuals [15]. Richards et al. revealed that serum adiponectin levels were negatively associated with BMD in postmenopausal women, even at non-load-bearing sites, suggesting that adiponectin affects bone metabolism through non-mechanical mechanisms [16]. Circulating leptin levels were previously reported to be elevated in obese individuals and positively correlated with BMD in humans [17]. The influences of leptin on bone metabolism are complicated by its enhancing and inhibitory effects on bone mass through peripheral and central actions, respectively, in mice [17]. On the other hand, Bartell et al. showed the contradictory findings that central leptin injection increases bone formation in leptin-deficient *ob/ob* mice, which is in agreement with previous studies that hypothalamic leptin gene therapy rescues a reduction in BMD in *ob/ob* mice [18–20]. As for muscle, leptin may exert anabolic effects on skeletal muscle in mice [21].

Muscle atrophy and osteopenia are concomitantly induced by a reduction of mechanical stress, such as long term space flight [22], which are prevented by exercise in astronauts [23], suggesting that mechanical stress is crucial for maintaining muscle and bone homeostasis. However, the effects of obesity on recovery from disuse-induced muscle and bone loss have not yet been elucidated in detail. Moreover, the roles of adipose tissue-derived humoral factors in the recovery of muscle and bone mass after reloading in unloading-induced sarcopenia and osteopenia remain unknown. Therefore, we herein examined the effects of obesity on recovery after reloading from disuse-induced muscle and bone loss using high fat diet (HFD)-fed mice with hindlimb unloading (HU) and subsequent reloading. Moreover, we aimed to investigate the roles of humoral factors from WAT on the increases observed in muscle and bone mass during mechanical reloading in mice fed HFD.

## Materials and methods

### Ethics statement

Animal experiments were performed according to the guidelines of the National Institutes of Health and the institutional rules for the use and care of laboratory animals at Kindai University. All animal experiments were approved by the Experimental Animal Welfare Committee of Kindai University (Permit number: KAME-27-029). All efforts were made to minimize suffering. Mice were euthanized with excess isoflurane.

### Animal experiments

Male C57BL/6J mice were obtained from CLEA Japan (Tokyo, Japan). Four-week-old mice were fed *ad libitum* with a normal diet (ND) or HFD (57% of calories from fat, CLEA Japan) and water. Mice were fed ND or HFD for 15 weeks, and HU was started at 8 weeks and reloading at 11 weeks after ND or HFD feeding was started. Mice were randomly divided into four groups: ND-fed control (ND/Control, n = 8), HFD-fed control (HFD/Control, n = 8), ND-fed reloading after HU (ND/reload, n = 8) and HFD-fed reloading after HU (HFD/reload, n = 8) groups. To induce HU, the mouse tail was suspended using a tail clip (Yamashita Giken, Tokushima, Japan) for 3 weeks at 8 weeks after ND or HFD feeding was started, as previously described [24]. HU mice were reloaded by removing the tail clip for 4 weeks after HU for 3 weeks. Mice with 6 hours of fasting were anesthetized using 2% isoflurane, and blood samples were collected 4 weeks after reloading. After mice were euthanized with excess isoflurane, epididymal and subcutaneous WAT were removed. In the present study, epididymal and subcutaneous WAT represent visceral and body fat, respectively. The ND/Control and HFD/Control groups were not subjected to HU followed by reloading.

### Quantitative computed tomography (QCT) analysis

A QCT analysis was performed using an X-ray CT system *in vivo* (Latheta LCT-200; Hitachi Aloka Medical, Tokyo, Japan) on mice 3 weeks after HU and then 4 weeks after reloading according to the guidelines of the American Society for Bone and Mineral Research [25], as described previously [24]. After mice were anesthetized with 2% isoflurane, CT images were acquired using the following parameters: 50 kVp tube voltage, 500 μA tube current, 48 mm axial field of view, and 96×192×1008 μm voxel size to analyze total fat and muscle masses, 48×48×192 μm voxel size to analyze fat and muscle masses in the lower leg, and 24 μm isotropic voxel size to analyze tibial BMD. The region of interest for the assessment of total fat and muscle masses was defined as the whole body. Regions of interest were defined as 1680-μm segments 96 μm distal to the end of the proximal growth plate towards the diaphysis for the assessment of trabecular BMD and as 2160-μm segments of the mid-diaphysis of the tibia for the assessment of cortical BMD. The region of interest for the assessment of fat and muscle masses in the lower leg was defined as the segment from the proximal to distal end of the tibia. Fat and muscle masses and BMD were analyzed using LaTheta software (version 3.41).

### Measurement of grip strength

The grip strength was measured using a grip strength meter (1027SM, Columbus Instruments, Columbus, OH, USA) 3 weeks after HU and then 4 weeks after reloading, as described previously [24]. Mice were allowed to grip a pull bar attachment by the four limbs. The mouse tail was then continuously pulled at a rate of approximately 2 cm/sec. The grip strength was measured five times and the results obtained were represented as an average for each mouse.

### Real-time PCR

Total RNA was isolated from mouse epididymal and subcutaneous WAT using Trizol reagent (Thermo Fisher Scientific, Waltham, MA, USA) and purified using an RNeasy Mini Kit (Qiagen, Hilden, Germany), as previously described [24]. The reverse transcription reaction was performed using a High-Capacity cDNA Reverse Transcription Kit (Applied Biosystems, Foster, CA, USA). cDNA was amplified by real-time PCR using ABI PRISM 7900HT (Applied Biosystems) with the Fast SYBR Green Master Mix (Applied Biosystems). Each PCR primer sequence is shown in S1 Table. Relative changes in target gene levels were calculated using the ΔΔCt method, and were normalized with 18S rRNA levels.

### Blood chemistry

Serum leptin levels were measured using the Quantikine enzyme-linked immunosorbent assay kit for mouse/rat leptin (R&D systems, Minneapolis, MN, USA, Cat. No. MOB00) [26]. Intra- and inter-assay coefficients of variations were ≤4.3% (n = 20) and ≤7.6% (n = 20), respectively.

### Statistical analysis

Data are represented as the mean ± standard error of the mean (SEM). Relative changes (% before reloading) in body weight, calorie intake, fat mass, BMD, muscle mass, and grip strength were calculated by dividing each parameter before reloading by that after reloading for 4 weeks. The significance of differences was evaluated using the Mann-Whitney *U* test for comparisons of 2 groups. A two-way analysis of variance followed by the Tukey-Kramer test was performed for multiple comparisons. Main effects (unloading and HFD, or reloading and HFD) were reported if there was no interaction effect. Pearson’s test was performed for a simple regression analysis. The significance level was set at *P* < 0.05. All statistical analyses were performed using GraphPad PRISM 7.00 software.

## Results

### Effects of HU and reloading on body weight and fat mass in mice fed HFD

**Fig 1:**
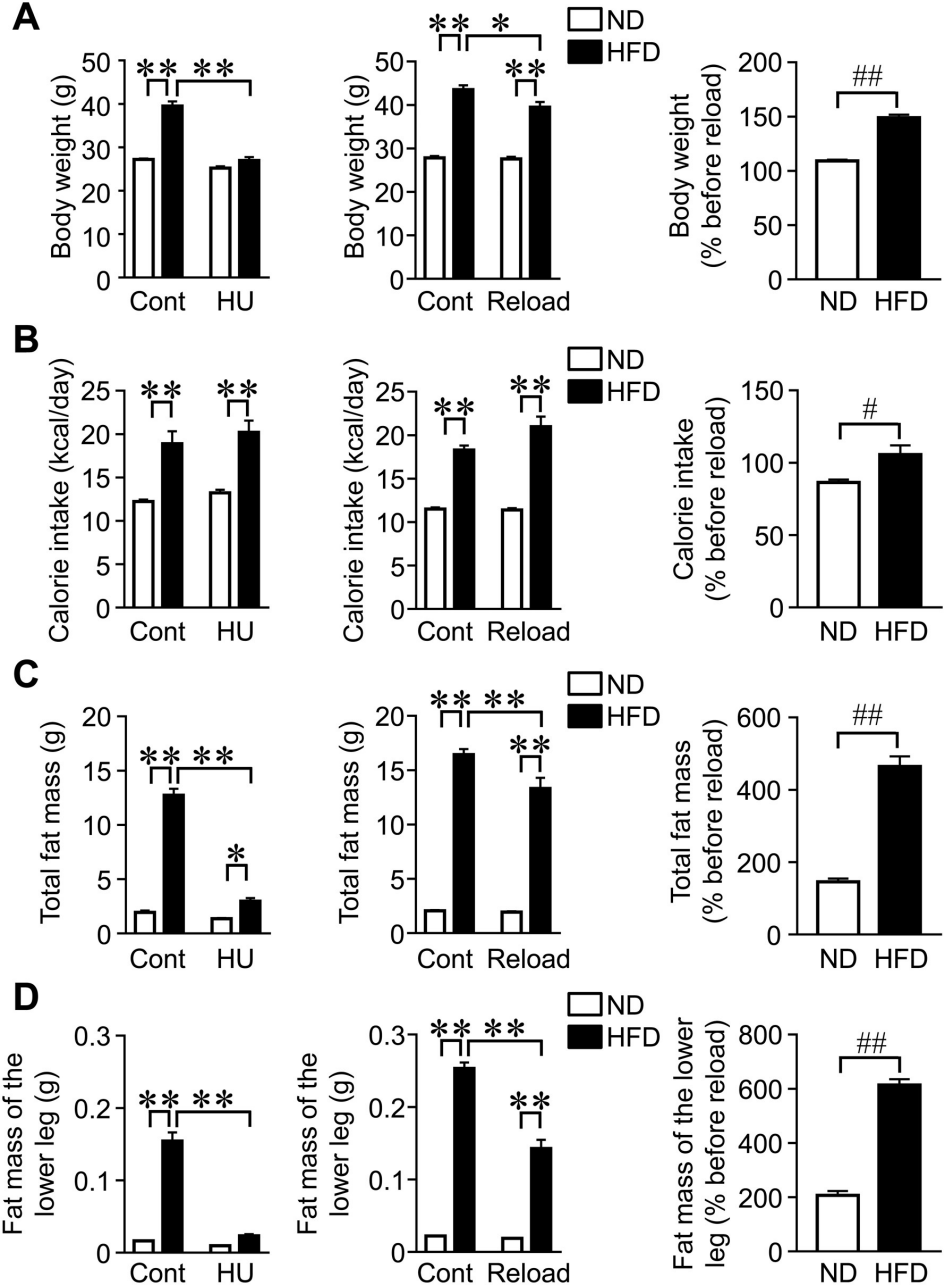
Effects of HU and reloading on body weight and fat mass in mice fed HFD. (A, B) Data on body weight and calorie intake from control (Cont), hindlimb unloading (HU), and reloading mice fed ND or HFD. Body weight was measured after HU for 3 weeks and subsequent reloading for 4 weeks. Food intake was collected for 3 days on days 18 to 21 after HU and days 25 to 28 after reloading, and shown as a representative of the average daily calorie intake. Relative changes were calculated by dividing body weight or calorie intake before reloading by those after reloading for 4 weeks. (C, D) Fat masses in the whole body and lower leg were assessed by QCT after HU for 3 weeks and subsequent reloading for 4 weeks in mice fed ND or HFD. Relative changes were calculated by dividing the total fat mass or fat mass in the lower leg before reloading by those after reloading for 4 weeks. *P < 0.05 and **P < 0.01 (Tukey-Kramer test). #*P* < 0.05 and ##*P* < 0.01 (Mann-Whitney *U* test). Data represent the mean ± SEM of 8 mice in each group.

There was a significant HFD × HU interaction in body weight (F_1,28_ = 53.8, *P* < 0.01). Body weights were significantly higher in mice fed HFD for 11 weeks than in mice fed ND (Fig 1A). HU for 3 weeks reduced body weight elevated by HFD in mice, whereas HU did not affect body weight in mice fed ND (Fig 1A). There was a significant HFD × reloading interaction in body weight (F_1,28_ = 5.11, *P* = 0.031). Body weights were significantly higher in mice fed HFD with reloading for 4 weeks than in reloading mice fed ND (Fig 1A). HFD significantly increased calorie intake in control and HU mice (Fig 1B), although there was no significant HFD × HU interaction (F_1,28_ = 0.024, *P* = 0.878). There was a significant HFD × reloading interaction in calorie intake (F_1,28_ = 4.499, *P* = 0.042, Fig 1B). Neither HU nor reloading affected calorie intake in mice fed ND and HFD (Fig 1B). There were no significant differences in the amount of food intake between control and HU mice fed HFD on day 18 to 21 after HU (Control, 3.7 ± 0.28 g/day; HU, 4.0 ± 0.27 g/day). There were significant interaction effects of HFD × HU on total fat mass (F_1,28_ = 176.3, *P* < 0.01), HFD × HU on fat mass of the lower leg (F_1,28_ = 105.2, *P* < 0.01), HFD × reloading on total fat mass (F_1,28_ = 7.173, *P* = 0.012), and HFD × reloading on fat mass of the lower leg (F_1,28_ = 53.38, *P* < 0.01). Fat masses in the whole body and lower leg were significantly higher in mice fed HFD than in mice fed ND, as assessed by the QCT analysis (Fig 1C and 1D). HU significantly reduced fat masses in the whole body and lower leg in mice fed HFD, but not in those fed ND (Fig 1C and 1D). HFD facilitated increases in fat masses in the whole body and lower leg in reloading mice over those in reloading mice fed ND (Fig 1C and 1D).

### Effects of HFD feeding on recovery from BMD reduced by HU after reloading in mice

**Fig 2:**
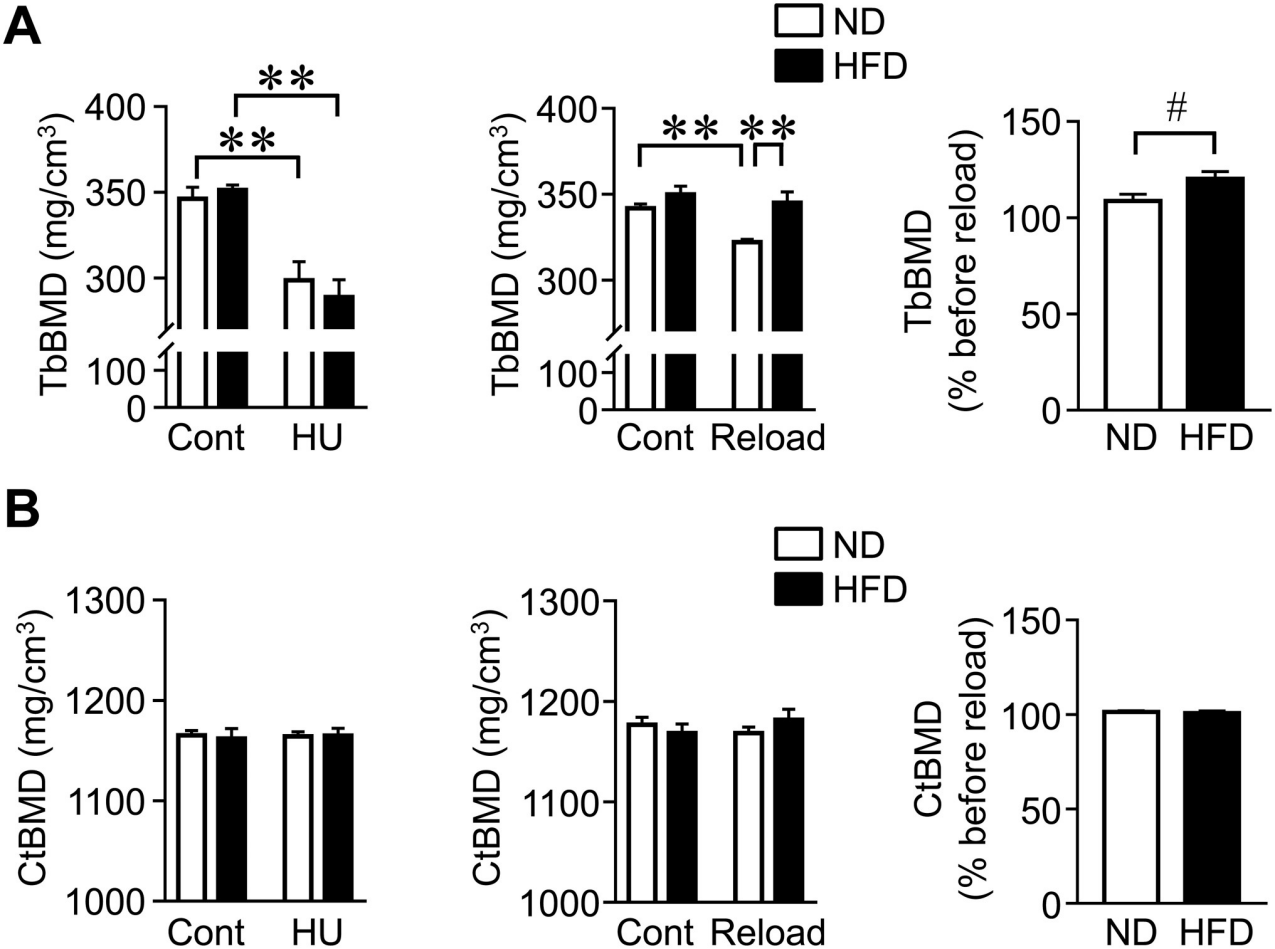
Effects of HU and reloading on trabecular and cortical BMDs in the tibia of mice fed HFD. (A, B) Trabecular (Tb) and cortical (Ct) BMDs in the tibia of mice fed ND or HFD were assessed by QCT after HU for 3 weeks and subsequent reloading for 4 weeks. Relative changes were calculated by dividing trabecular or cortical BMD before reloading by those after reloading for 4 weeks. **P < 0.01 (Tukey-Kramer test). #*P* < 0.05 (Mann-Whitney *U* test). Data represent the mean ± SEM of 8 mice in each group.

We examined the effects of HU and reloading on BMD in mice fed ND and HFD. There were no significant interaction effects of HFD × HU on trabecular BMD (F_1,28_ = 0.833, *P* = 0.369), HFD × reloading on trabecular BMD (F_1,28_ = 3.021, *P* = 0.093), HFD × HU on cortical BMD (F_1,28_ = 0.075, *P* = 0.786), and HFD × reloading on cortical BMD (F_1,28_ = 1.678, *P* = 0.206). HU significantly reduced trabecular BMD in the tibia of mice fed ND and HFD (Fig 2A). Although reloading increased trabecular BMD in the tibia of mice fed ND and HFD after HU, trabecular BMD was significantly higher in mice fed HFD than in mice fed ND (Fig 2A). Neither HU nor reloading affected cortical BMD in mice fed ND and HFD (Fig 2B).

### Effects of HFD feeding on recovery from reductions in muscle mass and grip strength by HU after reloading in mice

**Fig 3:**
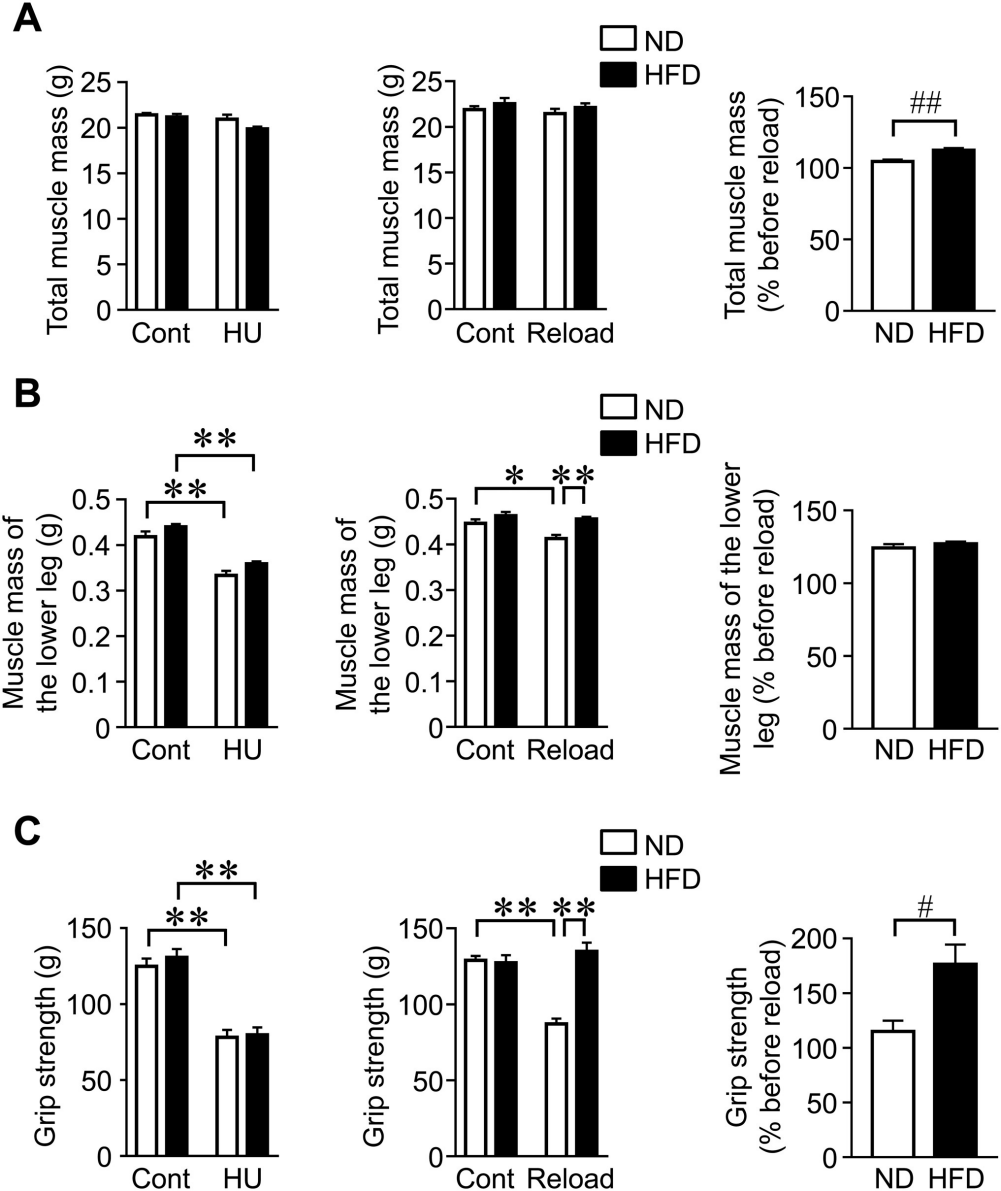
Effects of HU and reloading on muscle mass and grip strength in mice fed HFD. (A, B) Muscle masses in the whole body and lower leg were assessed by QCT after HU for 3 weeks and subsequent reloading for 4 weeks in mice fed ND or HFD. Relative changes were calculated by dividing the total muscle mass or muscle mass in the lower leg before reloading by those after reloading for 4 weeks. (C) The grip strength of four limbs was measured using a grip strength meter in mice fed ND or HFD after HU for 3 weeks and subsequent reloading for 4 weeks. Relative changes (% before reloading) in grip strength were calculated by dividing each parameter before reloading by that after reloading for 4 weeks. Data represent the mean ± SEM of 8 mice in each group. *P < 0.05 and **P < 0.01 (Tukey-Kramer test). #*P* < 0.05 and ##*P* < 0.01 (Mann-Whitney *U* test).

We examined the effects of HU and reloading on muscle mass and strength in mice fed ND and HFD. There were no significant interaction effects of HFD × HU on total muscle mass (F_1,28_ = 1.467, *P* = 0.236), HFD × reloading on total muscle mass (F_1,28_ = 0.001, *P* = 0.970), HFD × HU on muscle mass of the lower leg (F_1,28_ = 0.072, *P* = 0.821), and HFD × reloading on muscle mass of the lower leg (F_1,28_ = 2.919, *P* = 0.099). Muscle mass in the whole body was significantly reduced by HU (F_1,28_ = 6.907, *P* = 0.014), but not reloading, in mice fed ND or HFD (Fig 3A). The increase observed in muscle mass in the whole body was significantly higher in reloading mice fed HFD than in mice fed ND (Fig 3A). Muscle mass in the lower leg in mice was significantly reduced and increased by HU (F_1,28_ = 93.73, *P* < 0.01) and HFD (F_1,28_ = 7.475, *P* = 0.011), respectively (Fig 3B). Muscle mass in the lower leg was higher in reloading mice fed HFD than in mice fed ND (Fig 3B), although it was significantly increased by HFD (F_1,28_ = 15.02, *P* < 0.01). No significant differences were observed in the recovery rate of muscle mass in the lower leg between reloading mice fed ND and HFD (Fig 3B). HU significantly reduced grip strength in mice fed ND and HFD (Fig 3C), although there was no significant HFD × HU interaction (F_1,28_ = 0.176, *P* = 0.678). There was a significant HFD × reloading interaction in grip strength (F_1,28_ = 31.22, *P* < 0.01). HFD feeding significantly increased grip strength and its recovery rate in reloading mice over that in reloading mice fed ND (Fig 3C).

### Levels of humoral factors in epididymal and subcutaneous WAT in reloading mice fed ND and HFD

**Fig 4:**
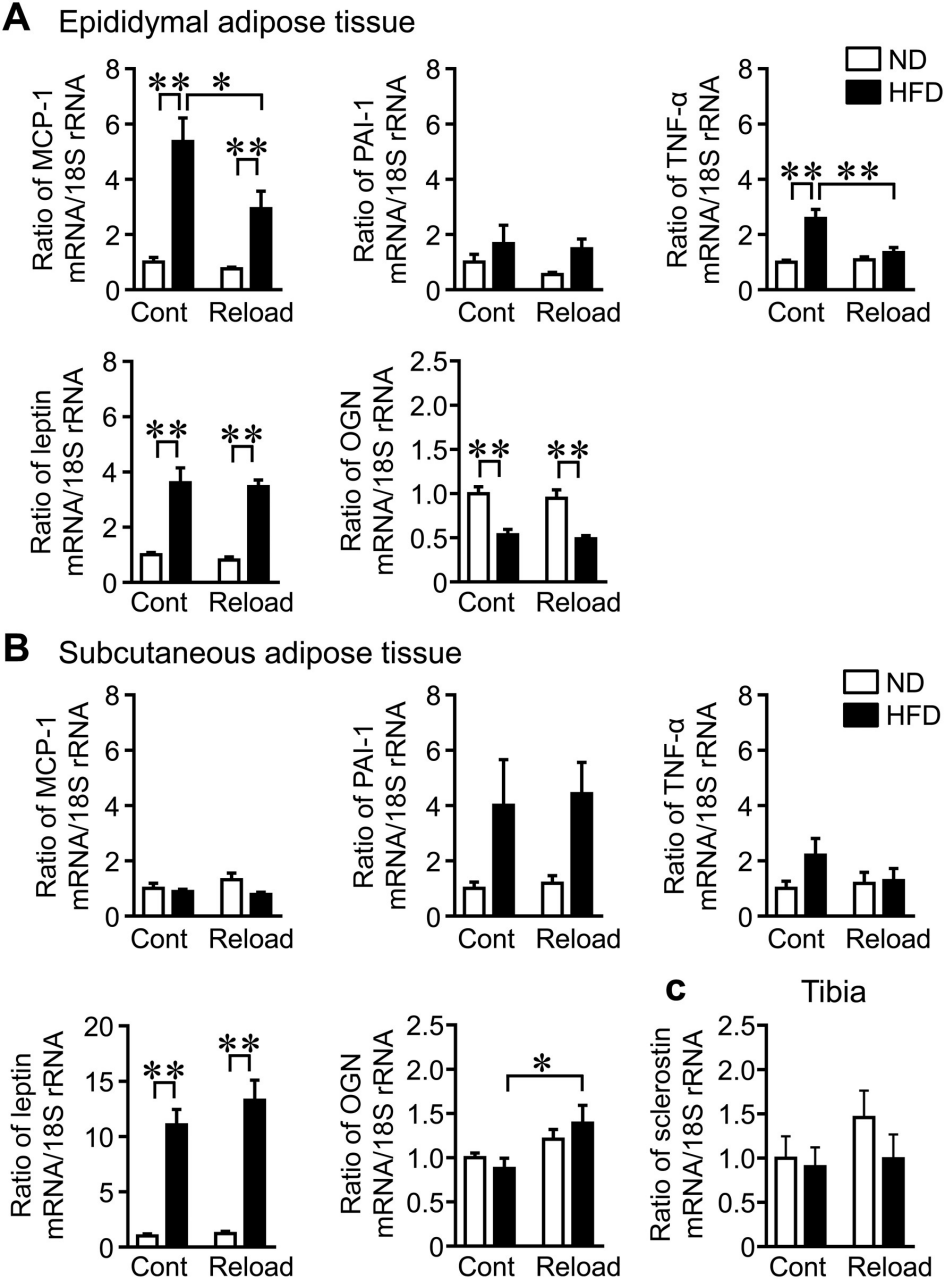
Effects of HU and reloading on mRNA levels of humoral factors in the epididymal and subcutaneous adipose tissue of mice fed HFD. (A, B) Total RNA was extracted from the epididymal (A) and subcutaneous (B) adipose tissue of mice fed ND or HFD after reloading for 4 weeks. A real-time PCR analysis of MCP-1, PAI-1, TNF-α, leptin, osteoglycin (OGN), or 18S rRNA was performed. (C) Total RNA was extracted from the tibia of mice fed ND or HFD after reloading for 4 weeks. A real-time PCR analysis of sclerostin or 18S rRNA was then performed. Data are expressed relative to 18S rRNA levels. Data represent the mean ± SEM from 8 mice in each group. *P < 0.05 and **P < 0.01 (Tukey-Kramer test).

Adipocytokines, such as MCP-1, PAI-1, TNF-α, and leptin, influence bone metabolism [13,14,17,21,27]. We therefore examined the levels of these adipocytokines in the epididymal and subcutaneous WAT of reloading mice fed ND and HFD. MCP-1 mRNA levels were significantly higher in the epididymal, but not subcutaneous, WAT of control and reloading mice fed HFD than in mice fed ND (Fig 4A and 4B), although there was no significant HFD × reloading interaction (F_1,28_ = 4.134, *P* = 0.052). PAI-1 mRNA levels were significantly increased by HFD in the subcutaneous WAT of control and reloading mice with no HFD × reloading interaction (Fig 4B). There was a significant HFD × reloading interaction on TNF-α mRNA levels in the epididymal WAT of mice (F_1,28_ = 11.11, *P* = 0.002). Although TNF-α mRNA levels were elevated in the epididymal WAT of control mice fed HFD, HFD feeding did not affect TNF-α mRNA levels in reloading mice (Fig 4A). Leptin mRNA levels in epididymal and subcutaneous WAT were significantly higher in mice fed HFD than in mice fed ND (Fig 4A and 4B), although there were no HFD × reloading interaction effects in epididymal (F_1,28_ = 0.008, *P* = 0.930) and subcutaneous (F_1,28_ = 0.748, *P* = 0.395) adipose tissues. Osteoglycin is a novel circulating humoral factor that affects bone metabolism and glucose homeostasis [28]. Osteoglycin levels are negatively related to fat and bone masses in mice fed HFD [28]. We therefore examined osteoglycin expression in the epididymal and subcutaneous WAT of mice fed ND and HFD. Osteoglycin mRNA levels were significantly lower and higher in epididymal and subcutaneous WAT, respectively, in mice fed HFD than in those fed ND with no HFD × reloading interaction (Fig 4A and 4B). Sclerostin, an inhibitor of Wnt/β-catenin signaling, was previously reported to be up-regulated in bone and suppressed bone formation during mechanical unloading [29]. We therefore investigated sclerostin expression in tibial bone tissues. HFD feeding did not affect sclerostin mRNA levels in the tibia of mice (Fig 4C).

### Relationships between the expression of humoral factors in WAT and the recovery rate of trabecular BMD in the tibia of reloading mice fed ND and HFD

**Fig 5:**
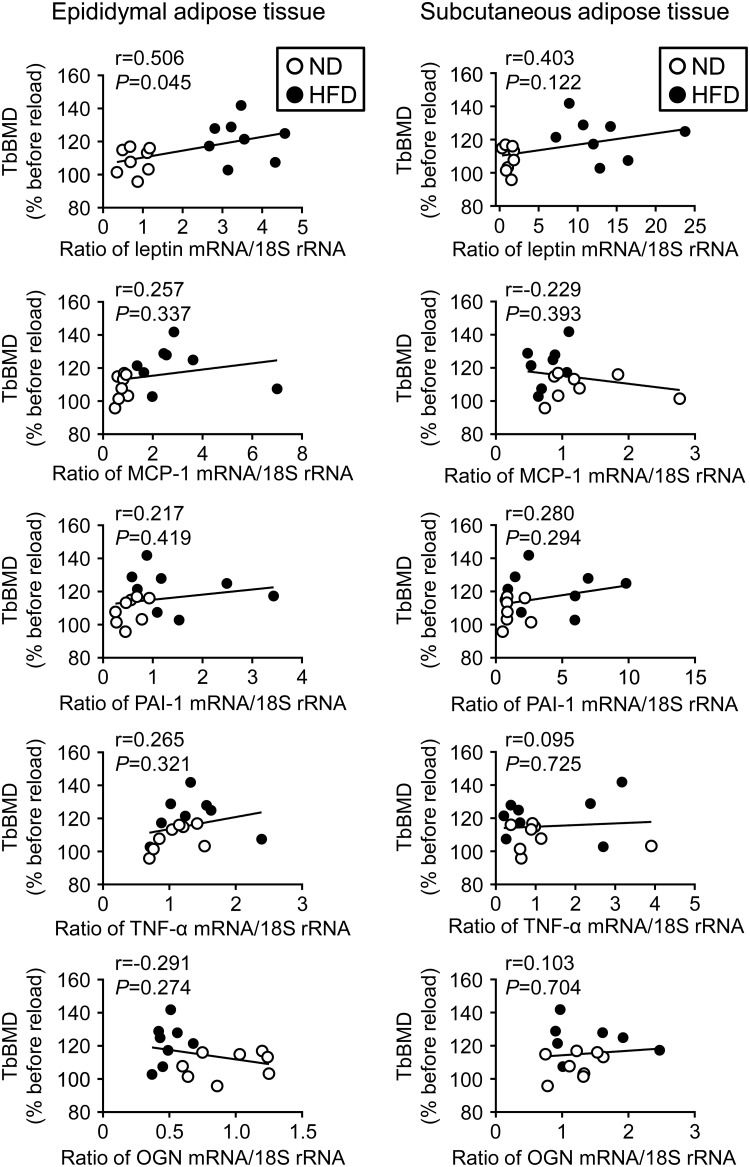
Relationships between humoral factor expression in adipose tissue and trabecular BMD in the tibia of mice fed ND or HFD. A simple regression analysis was performed on relative changes in trabecular BMD in the tibia and the mRNA levels of leptin, MCP-1, PAI-1, TNF-α, or osteoglycin (OGN) in the epididymal and subcutaneous adipose tissues of mice fed ND or HFD after reloading for 4 weeks. Relative changes (% before reloading) in BMD were calculated by dividing each parameter before reloading by that after reloading for 4 weeks. Data represent the mean ± SEM of 8 mice in each group.

We examined the relationships between the expression of humoral factors in WAT and the recovery rate of trabecular BMD in reloading mice using a simple regression analysis to identify which factors are involved in the enhanced recovery from HU-induced bone loss in obese mice. Leptin mRNA levels in epididymal WAT positively correlated with the recovery rate of trabecular BMD in reloading mice fed ND and HFD (Fig 5). Moreover, leptin mRNA levels in epididymal as well as subcutaneous WAT positively correlated with trabecular BMD in control and reloading mice fed ND and HFD (S2 Table). On the other hand, no significant relationships were observed between the recovery rate of trabecular BMD and the mRNA levels of MCP-1, PAI-1, TNF-α, and osteoglycin in the epididymal and subcutaneous WAT of reloading mice fed ND and HFD (Fig 5); however, the mRNA levels of MCP-1 and osteoglycin in epididymal WAT positively and negatively correlated with trabecular BMD in control and reloading mice fed ND and HFD, respectively (S2 Table). Sclerostin mRNA levels in tibial bone tissues did not correlate with the recovery rate of trabecular BMD in reloading mice fed ND and HFD (r = 0.235, *P* = 0.381).

### Relationships between humoral factors in WAT and the recovery rate of muscle mass and strength in reloading mice fed ND and HFD

We examined the relationships between the expression of humoral factors in WAT and the recovery rate of muscle mass and strength in mice fed ND and HFD. Leptin mRNA levels in epididymal and subcutaneous WAT positively correlated with the recovery rate of muscle mass in the whole body in mice fed ND and HFD (S3 Table). MCP-1 and PAI-1 mRNA levels in epididymal WAT positively correlated with the recovery rate of muscle mass in the whole body (S3 Table). On the other hand, osteoglycin mRNA levels negatively correlated with the recovery rate of muscle mass in the whole body (S3 Table). Regarding muscle strength, the recovery rate of grip strength positively correlated with leptin and PAI-1 mRNA levels in the epididymal and subcutaneous WAT of reloading mice fed ND and HFD (S4 Table). Osteoglycin mRNA levels in subcutaneous WAT positively correlated with the recovery rate of grip strength in reloading mice fed ND and HFD (S4 Table). Sclerostin mRNA levels in tibial bone tissues did not correlate with the recovery rate of total muscle mass or grip strength in reloading mice fed ND and HFD (Total muscle mass: r = -0.446, *P* = 0.083, grip strength: r = -0.396, *P* = 0.129).

### Relationships between serum leptin levels and the recovery rate of trabecular BMD, muscle mass in the whole body, and grip strength in reloading mice

**Fig 6:**
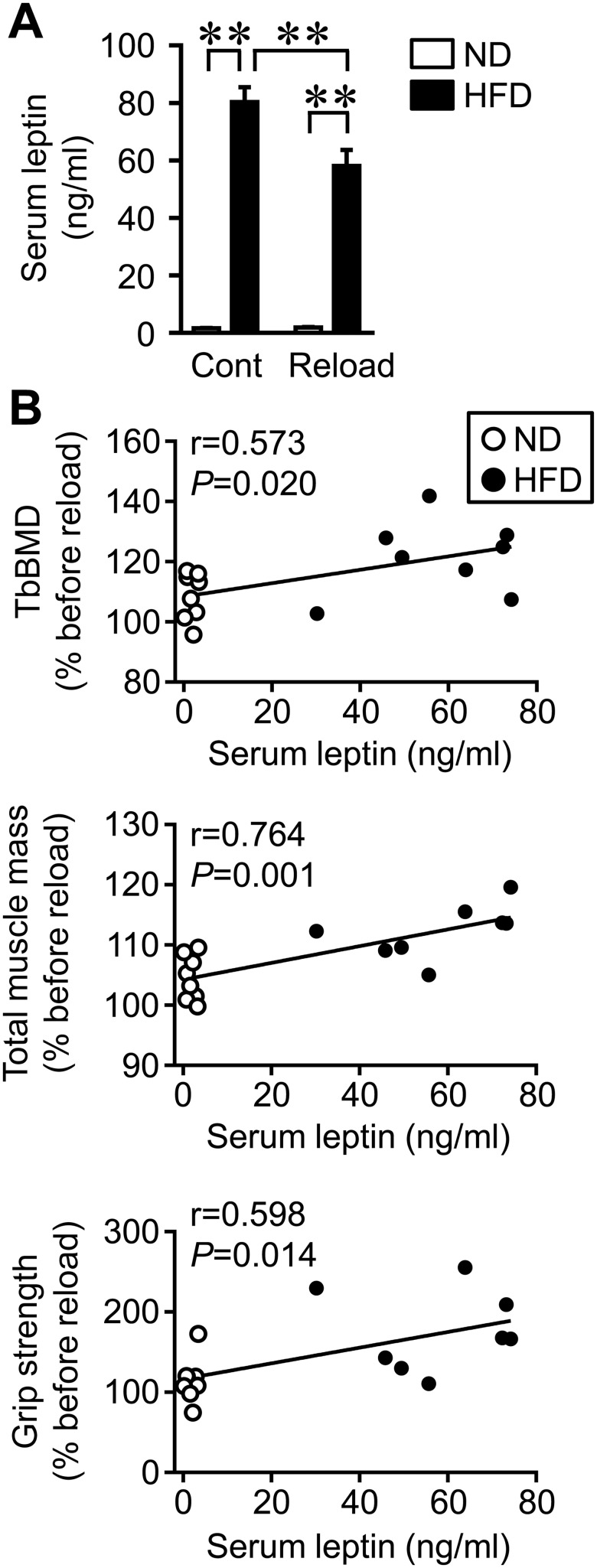
Relationships between serum leptin levels and trabecular BMD in the tibia, total muscle mass, and grip strength in mice fed ND or HFD. (A) Serum samples were collected from mice fed ND or HFD after reloading for 4 weeks. The quantification of serum leptin levels was then performed. **P < 0.01 (Tukey-Kramer test). (B) A simple regression analysis was performed on serum leptin levels and relative changes in trabecular BMD in the tibia, total muscle mass, or grip strength in mice fed ND or HFD after reloading for 4 weeks. Relative changes (% before reloading) in BMD, muscle mass, and grip strength were calculated by dividing each parameter before reloading by that after reloading for 4 weeks. Data represent the mean ± SEM of 8 mice in each group.

Since leptin mRNA levels in the WAT of reloading mice positively correlated with the recovery rate of trabecular BMD, muscle mass in the whole body, and grip strength, we investigated serum leptin levels in control and reloading mice fed ND and HFD. HFD feeding significantly increased serum leptin levels in control and reloading mice over those in mice fed ND (Fig 6A), although there was a significant HFD × reloading interaction (F_1,28_ = 8.722, *P* = 0.006). Serum leptin levels positively correlated with the recovery rate of trabecular BMD, muscle mass in the whole body, and grip strength in reloading mice fed ND and HFD (Fig 6B). Trabecular BMD, muscle mass in the lower leg, and grip strength positively correlated with fat mass in the whole body in control and reloading mice fed ND and HFD (S5 Table). Muscle mass in the whole body and grip strength positively correlated with the rate of increases in whole body fat mass (S5 Table).

## Discussion

Extensive clinical evidence has indicated that obese subjects have higher BMD than non-obese subjects, presumably through the increased mechanical stress caused by the higher body weight burden [4,7]. In the present study, obesity elevated trabecular BMD in reloading mice, but did not affect a reduction in trabecular BMD in HU mice. These findings were consistent with the recent evidence that obesity does not affect HU-induced bone loss in leptin-deficient *ob/ob* mice [30]. Previous studies revealed that the weight bearing resistance exercise using weighted vests increases trabecular bone area and BMD in rats fed ND [31,32]. Song et al. showed that resistance ladder climbing exercise with weight bearing facilitates the recovery from a reduction in BMD by HU after reloading in rats [33]. These findings suggest that HFD facilitates the recovery of bone mass reduced by mechanical unloading after reloading partly through an enhancement of mechanical stress by the increased body weight. As for the skeletal muscles, we showed that HFD elevates muscle mass in the lower leg and grip strength in reloading mice in the present study, but did not affect the reductions in those muscle-related parameters in HU mice. We therefore cannot rule out the possibility that HFD may facilitate the recovery of bone mass reduced by mechanical unloading after reloading partly through a mechanical stimulation by increased muscle mass and strength on bone in mice.

In the present study, HFD increased the recovery rate of grip strength in reloading mice over that in reloading mice fed ND, although the effects of HFD on the recovery rate of muscle mass seemed to be less. These results suggest that the recovery of muscle function is not only attributable to recovery of muscle mass after reloading. It may be due to the greater body weight or muscle loading. Alternatively, a previous study showed that HU impairs neuromuscular axonal excitability in mice [34]. Avraham et al. showed that leptin treatment exerts neruoprotective effects through enhancement of neurogenesis and angiogenesis after stroke in mice [35]. These findings suggest that that improved neural activation may be partly involved in the recovery of muscle function reduced by HU after reloading in mice fed HFD.

Adipocytokines produced from adipose tissues in the obese state play crucial roles in the regulation of bone and muscle in obesity [1,11]. Therefore, some adipocytokines induced by the obese state may be related to the recovery of bone mass, muscle mass, and grip strength reduced by mechanical unloading after reloading in mice. Leptin regulates bone metabolism through central and peripheral actions [17]. Previous studies on mice reported that leptin suppressed and enhanced bone formation and bone resorption, respectively, through central actions [17,36], whereas Bartell et al. showed contradictory findings on the central actions of leptin [18]. On the other hand, previous studies indicate that leptin enhances and suppresses bone formation and resorption, respectively, through peripheral action *in vitro* [37,38]. Indeed, numerous clinical studies indicated that leptin exerts positive effects on bone in humans [17,39]. Yamauchi et al. revealed that circulating leptin levels were positively and inversely related to BMD at the lumbar spine, femoral neck, and forearm as well as the presence of vertebral fractures, respectively, in postmenopausal women [39]. Roux et al. showed that circulating leptin levels were positively related to BMD at the lumbar spine and femoral neck in healthy postmenopausal women [40]. In the present study, adipose tissue leptin expression and serum leptin levels were elevated in mice fed HFD. Moreover, the recovery rate of trabecular BMD after reloading was positively related to adipose tissue leptin expression and serum leptin levels in mice. Collectively, these findings and the present results suggest that leptin contributes to the obesity-induced increased trabecular bone mass presumably through peripheral action. Regarding the effects of leptin on skeletal muscle, leptin enhanced the proliferation and myogenic differentiation of primary myoblasts [41]. In the study by Sáinz et al., leptin treatment increased muscle mass and reduced the levels of the protein degradation-related factor, muscle RING-finger protein-1, in mice [42]. In the present study, serum leptin levels were elevated in mice fed HFD, and positively correlated with the recovery rates of muscle mass and strength in reloading mice. These findings suggest that elevated circulating leptin levels are involved in the recovery of muscle mass and strength enhanced by obesity in reloading mice. However, although leptin mRNA levels in the epididymal adipose tissues were positively related to the recovery rate of trabecular BMD with statistical significance in the present study, the correlation was not strong, suggesting that leptin is partly responsible for the recovery of BMD enhanced by obesity.

MCP-1, a chemoattractant for monocytes and macrophages, plays a key role in obesity-associated pathological conditions [43]. In the present study, MCP-1 expression was elevated in the epididymal adipose tissue of mice fed HFD. Moreover, MCP-1 expression in epididymal adipose tissues positively correlated with an increase in muscle mass, but not muscle strength, in reloading mice. Wang et al. reported that inflammatory macrophages impair muscle differentiation in obese mice [10]. Moreover, circulating MCP-1 levels were higher in obese individuals with or without sarcopenia than in non-obese individuals [44]. These findings suggest that an elevation in MCP-1 levels in epididymal adipose tissues is not responsible for muscle mass recovery enhanced by obesity in reloading mice. Although PAI-1, a primary inhibitor of plasminogen activators, plays pleiotropic roles as an adipokines and negatively regulates muscle mass and functions [12,14,45], PAI-1 expression in the adipose tissues was positively related to the recovery rates of muscle mass and strength in reloading mice. Therefore, PAI-1 may not be responsible for the facilitation of muscle mass and strength recovery induced by obesity in reloading mice. As for osteoglycin, a class III small leucine-rich proteoglycan, its expression in the epididymal adipose tissues was reduced by HFD and negatively correlated with the recovery rates of muscle mass and strength by reloading in mice in our study, although it may exert positive effects on muscle mass [28,46]. Further studies will be necessary to clarify the roles of osteoglycin in the recovery process of skeletal muscle in an obese state.

The mechanisms by which HU reduced fat mass enhanced by HFD have remained unknown in the present study. HU did not affect calorie intake in mice fed ND and HFD. Moreover, there were no significant differences in the amount of food intake between control and HU mice fed HFD on days 18 to 21 after HU, which is consistent with previous studies in leptin-deficient *ob/ob* mice [30]. These results suggest that a decrease in fat mass in mice fed HFD is not attributable to amounts of calorie and food intakes during HU. Since HU reduces bone mass through the sympathetic nervous system, which stimulates lipolysis [47,48], an activation of the sympathetic nervous system by HU may decrease fat mass in HFD mice.

There were several limitations in the present study. First, the direct biological relationships between leptin and the recovery of bone and muscle after reloading have still remained unclear, although we showed the significant relationships between serum leptin levels and the increases in trabecular BMD, total muscle mass, and grip strength after reloading in mice using a simple regression analysis. Further studies using an anti-leptin neutralizing antibody for mice, leptin-deficient *ob/ob*, and leptin receptor-deficient *db/db* mice will be necessary to clarify these issues. Next, there are contradictory findings due to differences in nutrient composition, sources of fats, and percentage of fats, although high-fat diet-induced obesity in mice is well established as an animal model of obesity [49,50]. HFD used in the present study does not appear to reflect a normal human diet which typically contains <35% of calories from fat [49].

Sarcopenic obesity is a multifactorial condition and characterized by comorbidity of sarcopenia and obesity [51]. Sarcopenia may be exacerbated by the presence of obesity. Although effective treatments for sarcopenia have not been established yet, the exercise therapy may be effective for sarcopenia [52]. The present findings suggest that obesity facilitates the recovery of muscle and bone reduced by HU after reloading in mice. We can therefore speculate that exercise may be more effective in patients with sarcopenic obesity, compared to those with sarcopenia without obesity.

In conclusion, the present results provide novel evidence to show that obesity enhances the recovery of bone and muscle masses as well as strength decreased by mechanical unloading after reloading in mice. Our data suggested that leptin may be related to the recovery of muscle and bone enhanced by obesity in mice; further studies are required to elucidate the mechanisms by which the reloading induces the recovery of muscle and bone in mice fed HFD. Leptin may be a target for the prevention and treatment of immobilization-induced osteoporosis and sarcopenia.

## Supporting information

S1 Table Primers used in real-time PCR experiments. MCP-1, monocyte chemoattractant protein-1; PAI-1, plasminogen activator inhibitor-1; TNF-α, tumor necrosis factor-α.
(DOCX)

S2 Table Relationship between trabecular BMD in the tibia and humoral factors in the adipose tissue of mice fed ND or HFD. A simple regression analysis was performed on trabecular BMD in the tibia and mRNA levels of leptin, MCP-1, PAI-1, TNF-α, or osteoglycin in the epididymal and subcutaneous adipose tissues of mice fed ND or HFD after reloading for 4 weeks. MCP-1, monocyte chemoattractant protein-1; PAI-1, plasminogen activator inhibitor-1; TNF, tumor necrosis factor.
(DOCX)

S3 Table Relationship between total muscle mass and humoral factors in the adipose tissue of mice fed ND or HFD. A simple regression analysis was performed on total muscle mass or its relative changes and the mRNA levels of leptin, MCP-1, PAI-1, TNF-α, or osteoglycin in the epididymal and subcutaneous adipose tissue of mice fed ND or HFD after reloading for 4 weeks. MCP-1, monocyte chemoattractant protein-1; PAI-1, plasminogen activator inhibitor-1; TNF, tumor necrosis factor.
(DOCX)

S4 Table Relationship between grip strength and humoral factors in the adipose tissue of mice fed ND or HFD. A simple regression analysis was performed on grip strength or its relative changes and the mRNA levels of leptin, MCP-1, PAI-1, TNF-α, or osteoglycin in the epididymal and subcutaneous adipose tissue of mice fed ND or HFD after reloading for 4 weeks. MCP-1, monocyte chemoattractant protein-1; PAI-1, plasminogen activator inhibitor-1; TNF, tumor necrosis factor.
(DOCX)

S5 Table Relationship between total fat mass and parameters of bone and muscle in mice fed ND and HFD. A simple regression analysis was performed on total fat mass or its relative changes and trabecular BMD, cortical BMD, total muscle mass, muscle mass in the lower leg, or grip strength in mice fed ND or HFD after reloading for 4 weeks.
(DOCX)
