# Supplementary material for: Roles of leptin in the recovery of muscle and bone by reloading after mechanical unloading in high fat diet-fed obese mice
Source: PLoS One. 2019 Oct 24;14(10):e0224403. doi: 10.1371/journal.pone.0224403 (PMC6812756; doi:10.1371/journal.pone.0224403)
Supplement: S1 Table — MCP-1, monocyte chemoattractant protein-1; PAI-1, plasminogen activator inhibitor-1; TNF-α, tumor necrosis factor-α. (DOCX) [file pone.0224403.s001.docx]

**S1 Table.** Primers used in real-time PCR experiments.

| Gene |  | Primer sequence |
| --- | --- | --- |
| MCP-1 | Forward  Reverse | 5’-CCACTCACCTGCTGCTACTCA-3’  5’-TGGTGATCCTCTTGTAGCTCTCC-3’ |
| PAI-1 | Forward  Reverse | 5’-TTCAGCCCTTGCTTGCCTC-3’  5’-ACACTTTTACTCCGAAGTCGGT-3’ |
| TNF-α | Forward  Reverse | 5’-CCCAGACCCTCACACTCAGATC-3’  5’-GCCACTCCAGCTGCTCCTC-3’ |
| Leptin | Forward  Reverse | 5’-TGACACCAAAACCCTCATCA-3’  5’-AGCCCAGGAATGAAGTCCA-3’ |
| Osteoglycin  Sclerostin | Forward  Reverse  Forward  Reverse | 5’-TGCTTTGTGGTCACATGGAT-3’  5’-GAAGCTGCACACAGCACAAT-3’  5’-CTACTTGTGCACGCTGCCTT-3’  5’-TTTGGCGTCATAGGGATGGT-3’ |
| 18S rRNA | Forward | 5’-CGGCTACCACATCCAAGGAA-3’ |
|  | Reverse | 5’-GCTGGAATTACCGCGGCT-3’ |

MCP-1, monocyte chemoattractant protein-1; PAI-1, plasminogen activator inhibitor-1; TNF-α, tumor necrosis factor-α.
