# Supplementary material for: Roles of leptin in the recovery of muscle and bone by reloading after mechanical unloading in high fat diet-fed obese mice
Source: PLoS One. 2019 Oct 24;14(10):e0224403. doi: 10.1371/journal.pone.0224403 (PMC6812756; doi:10.1371/journal.pone.0224403)
Supplement: S2 Table — A simple regression analysis was performed on trabecular BMD in the tibia and mRNA levels of leptin, MCP-1, PAI-1, TNF-α, or osteoglycin in the epididymal and subcutaneous adipose tissues of mice fed ND or HFD after reloading for 4 weeks. MCP-1, monocyte chemoattractant protein-1; PAI-1, plasminogen activator inhibitor-1; TNF, tumor necrosis factor. (DOCX) [file pone.0224403.s002.docx]

**S2 Table.** Relationship between trabecular BMD in the tibia and humoral factors in the adipose tissue of mice fed ND or HFD.

| Trabecular BMD | | | | | | |
| --- | --- | --- | --- | --- | --- | --- |
|  | Epididymal | |  |  | Subcutaneous | |
| Gene | r | *P* |  |  | r | *P* |
| Leptin | 0.453 | 0.009 |  |  | 0.482 | 0.005 |
| MCP-1 | 0.389 | 0.028 |  |  | -0.256 | 0.157 |
| PAI-1 | 0.233 | 0.199 |  |  | 0.230 | 0.206 |
| TNF-α | 0.243 | 0.180 |  |  | 0.245 | 0.176 |
| Osteoglycin | -0.475 | 0.006 |  |  | 0.174 | 0.342 |

A simple regression analysis was performed on trabecular BMD in the tibia and mRNA levels of leptin, MCP-1, PAI-1, TNF-α, or osteoglycin in the epididymal and subcutaneous adipose tissues of mice fed ND or HFD after reloading for 4 weeks. MCP-1, monocyte chemoattractant protein-1; PAI-1, plasminogen activator inhibitor-1; TNF, tumor necrosis factor.
