# Supplementary material for: Roles of leptin in the recovery of muscle and bone by reloading after mechanical unloading in high fat diet-fed obese mice
Source: PLoS One. 2019 Oct 24;14(10):e0224403. doi: 10.1371/journal.pone.0224403 (PMC6812756; doi:10.1371/journal.pone.0224403)
Supplement: S3 Table — A simple regression analysis was performed on total muscle mass or its relative changes and the mRNA levels of leptin, MCP-1, PAI-1, TNF-α, or osteoglycin in the epididymal and subcutaneous adipose tissue of mice fed ND or HFD after reloading for 4 weeks. MCP-1, monocyte chemoattractant protein-1; PAI-1, plasminogen activator inhibitor-1; TNF, tumor necrosis factor. (DOCX) [file pone.0224403.s003.docx]

**S3 Table.** Relationship between total muscle mass and humoral factors in the adipose tissue of mice fed ND or HFD.

|  | Total muscle mass | | | | |  | Total muscle mass  (% before reloading) | | | | |
| --- | --- | --- | --- | --- | --- | --- | --- | --- | --- | --- | --- |
|  | Epididymal | |  | Subcutaneous | |  | Epididymal | |  | Subcutaneous | |
| Gene | r | *P* |  | r | *P* |  | r | *P* |  | r | *P* |
| Leptin  MCP-1  PAI-1  TNF-α  Osteoglycin | 0.076  0.220  0.062  0.301  -0.375 | 0.680  0.226  0.737  0.094  0.034 |  | 0.317  0.219  0.222  -0.221  0.186 | 0.077  0.228  0.221  0.224  0.307 |  | 0.702  0.686  0.499  0.242  -0.617 | 0.002  0.003  0.049  0.367  0.011 |  | 0.746  -0.313  0.452  -0.237  0.243 | 0.001  0.238  0.079  0.378  0.364 |

A simple regression analysis was performed on total muscle mass or its relative changes and the mRNA levels of leptin, MCP-1, PAI-1, TNF-α, or osteoglycin in the epididymal and subcutaneous adipose tissue of mice fed ND or HFD after reloading for 4 weeks. MCP-1, monocyte chemoattractant protein-1; PAI-1, plasminogen activator inhibitor-1; TNF, tumor necrosis factor.
