# Supplementary material for: Roles of leptin in the recovery of muscle and bone by reloading after mechanical unloading in high fat diet-fed obese mice
Source: PLoS One. 2019 Oct 24;14(10):e0224403. doi: 10.1371/journal.pone.0224403 (PMC6812756; doi:10.1371/journal.pone.0224403)
Supplement: S4 Table — A simple regression analysis was performed on grip strength or its relative changes and the mRNA levels of leptin, MCP-1, PAI-1, TNF-α, or osteoglycin in the epididymal and subcutaneous adipose tissue of mice fed ND or HFD after reloading for 4 weeks. MCP-1, monocyte chemoattractant protein-1; PAI-1, plasminogen activator inhibitor-1; TNF, tumor necrosis factor. (DOCX) [file pone.0224403.s004.docx]

**S4 Table.** Relationship between grip strength and humoral factors in the adipose tissue of mice fed ND or HFD.

|  | Grip strength | | | | |  | Grip strength (% before reloading) | | | | |
| --- | --- | --- | --- | --- | --- | --- | --- | --- | --- | --- | --- |
|  | Epididymal | |  | Subcutaneous | |  | Epididymal | |  | Subcutaneous | |
| Gene | r | *P* |  | r | *P* |  | r | *P* |  | r | *P* |
| Leptin | 0.349 | 0.049 |  | 0.526 | 0.002 |  | 0.524 | 0.037 |  | 0.584 | 0.017 |
| MCP-1 | 0.302 | 0.092 |  | -0.383 | 0.030 |  | 0.336 | 0.203 |  | -0.333 | 0.207 |
| PAI-1 | 0.329 | 0.066 |  | 0.336 | 0.060 |  | 0.690 | 0.003 |  | 0.507 | 0.045 |
| TNF-α | 0.168 | 0.358 |  | 0.119 | 0.516 |  | -0.023 | 0.932 |  | 0.093 | 0.731 |
| Osteoglycin | -0.396 | 0.025 |  | 0.152 | 0.406 |  | -0.429 | 0.097 |  | 0.546 | 0.029 |

A simple regression analysis was performed on grip strength or its relative changes and the mRNA levels of leptin, MCP-1, PAI-1, TNF-α, or osteoglycin in the epididymal and subcutaneous adipose tissue of mice fed ND or HFD after reloading for 4 weeks. MCP-1, monocyte chemoattractant protein-1; PAI-1, plasminogen activator inhibitor-1; TNF, tumor necrosis factor.
