# Supplementary material for: Roles of leptin in the recovery of muscle and bone by reloading after mechanical unloading in high fat diet-fed obese mice
Source: PLoS One. 2019 Oct 24;14(10):e0224403. doi: 10.1371/journal.pone.0224403 (PMC6812756; doi:10.1371/journal.pone.0224403)
Supplement: S5 Table — A simple regression analysis was performed on total fat mass or its relative changes and trabecular BMD, cortical BMD, total muscle mass, muscle mass in the lower leg, or grip strength in mice fed ND or HFD after reloading for 4 weeks. (DOCX) [file pone.0224403.s005.docx]

**S5 Table.** Relationship between total fat mass and parameters of bone and muscle in mice fed ND and HFD.

|  | Total fat mass | |  | Total fat mass  (% before reloading) | |
| --- | --- | --- | --- | --- | --- |
|  | r | *P* |  | r | *P* |
| TbBMD | 0.497 | 0.004 |  | 0.441 | 0.087 |
| CtBMD | 0.035 | 0.850 |  | -0.136 | 0.617 |
| Total muscle mass | 0.308 | 0.087 |  | 0.766 | 0.001 |
| Muscle mass in the lower leg | 0.546 | 0.001 |  | 0.312 | 0.239 |
| Grip strength | 0.474 | 0.006 |  | 0.649 | 0.007 |

A simple regression analysis was performed on total fat mass or its relative changes and trabecular BMD, cortical BMD, total muscle mass, muscle mass in the lower leg, or grip strength in mice fed ND or HFD after reloading for 4 weeks.
